# Hospital Tablets and Memorials

**Published:** 1923-11

**Authors:** 


### HOSPITAL TABLETS AND MEMORIALS.

Inscriptions, tablets and memorials, like the portraits that enrich the board rooms of all well-managed voluntary hospitals, are valuable because of the traditions that they record and so preserve. From tradition, institutions, like men, draw their deepest life, but it is not always remembered that the hospital standard applies equally to these as to every other detail of institutional activity. A debased lettering upon a hospital slab or tablet is no less an error that reflects upon the management's standard than an ill-written letter, poor equipment, or incivility to patients and their friends is known to be. It is, therefore, well to remind those responsible for these recurrent necessities that three disinterested bodies now exist to give expert advice upon them. The Civic Arts Association has done valuable service in regard to war memorials; the Church Crafts League enters a kindred field, and the English Crafts and Monumental Society not only advises, free of charge, but can carry out memorials also. We have seen with gratitude the recovery of the beautiful old tradition of lettering that two of these bodies set out to achieve, and in the interest of the hospital standard in small things, as in great, have no hesitation in recommending others to acquaint themselves with it.

**Sussex County Hospital.**

Mr. Harry Preston has raised £15,000 for the Royal Sussex County Hospital, at Brighton, and the wards, lately closed, have been reopened.

